# The Effect of Cranial Sutures Should Be Considered in Transcranial Electrical Stimulation

**DOI:** 10.1097/YCT.0000000000001079

**Published:** 2024-12-10

**Authors:** Alistair Carroll, Caroline D. Rae, Donel Martin, Socrates Dokos, Colleen Loo

**Affiliations:** From the ∗ Discipline of Psychiatry, University of New South Wales, Sydney Australia; † University of New South Wales, Sydney, Australia.

**Keywords:** cranial suture conductance, transcranial electrical stimulation, EEG, computational modeling

## Abstract

**Background:**

Computational modeling is used to optimize transcranial electrical stimulation (tES) approaches, and the precision of these models is dependent on their anatomical accuracy. We are unaware of any computational modeling of tES that has included cranial sutures.

**Objectives:**

The aims of the study were to review the literature on the timing of closure of the coronal and squamous sutures, which are situated under electrode placements used in tES; to review the literature regarding differences in skull and suture conductivity and to determine a more accurate conductivity for sutures; and to identify magnetic resonance image (MRI) techniques that could be used to detect cranial sutures.

**Methods:**

A scoping review of medical literature was conducted. We conducted computational modeling of a cranial bone plug using COMSOL Multiphysics finite element software, utilizing methodology and results from a previous study. We assessed use of the “3D Slicer” software to identify sutures in routine T1-weighted MRI scans.

**Results:**

Reports from forensic examinations and computed tomography (CT) scans showed suture closure does not correlate with age. Our computational modeling determined a cranial suture conductivity of 0.32 S/m, which is much higher than for skull (compact skull 0.004 S/m, standard trilayer 0.013 S/m). 3D slicer enabled rapid and precise identification of the anatomy and location of cranial sutures.

**Conclusions:**

Cranial sutures persist throughout the lifespan and have a far higher conductivity than skull bone. Cranial sutures can be localized quickly and precisely using a combination of MRI and readily available modeling software. Sutures should be included in tES computational modeling and electroencephalography source imaging to improve the accuracy of results.

Transcranial electrical stimulation (tES) includes electroconvulsive therapy (ECT), transcranial direct current stimulation (tDCS), and other emerging forms of stimulation. ECT involves the administration of pulsed electrical current to induce generalized seizures and has been demonstrated to be more effective than pharmacotherapy for major depressive disorder.^1^ A recent Cochrane review concluded that there is moderate-quality evidence suggesting that it has a positive benefit in treatment-resistant schizophrenia.^2^ tDCS induces a constant, low-intensity, unidirectional current that has evidence of efficacy in the treatment of depression and emerging evidence in other disorders.^3^ Over recent decades, computational modeling has increasingly been used to inform tES treatment approach and electroencephalography (EEG) source imaging. Computational models need to be anatomically accurate to provide clinically relevant results and emerging imaging techniques will facilitate efficient and precise tissue segmentation to assist with this.

The term suture is coined from the Latin word “sutura,” referring to serrated fibrous interdigitations. There are 4 major sutures in the human skull. The coronal suture can be found between the frontal and parietal bones in the coronal plane, the lambdoid suture borders the parietal and occipital bones in the coronal plane, and the sagittal suture lies between the coronal and lambdoid sutures along the sagittal plane. The other major cranial suture is the squamous suture, in which digitations overlap like tiles on a roof. It is located bilaterally in the temporal bone (Fig. 1). Ectocranial (external) sutures can increase in complexity with age while endocranial (internal) sutures tend to remain in simple straight lines.^4^ Cranial sutures are often assumed to fuse in the early years of life and have been used forensically to determine the age of skulls.

**FIGURE 1:**
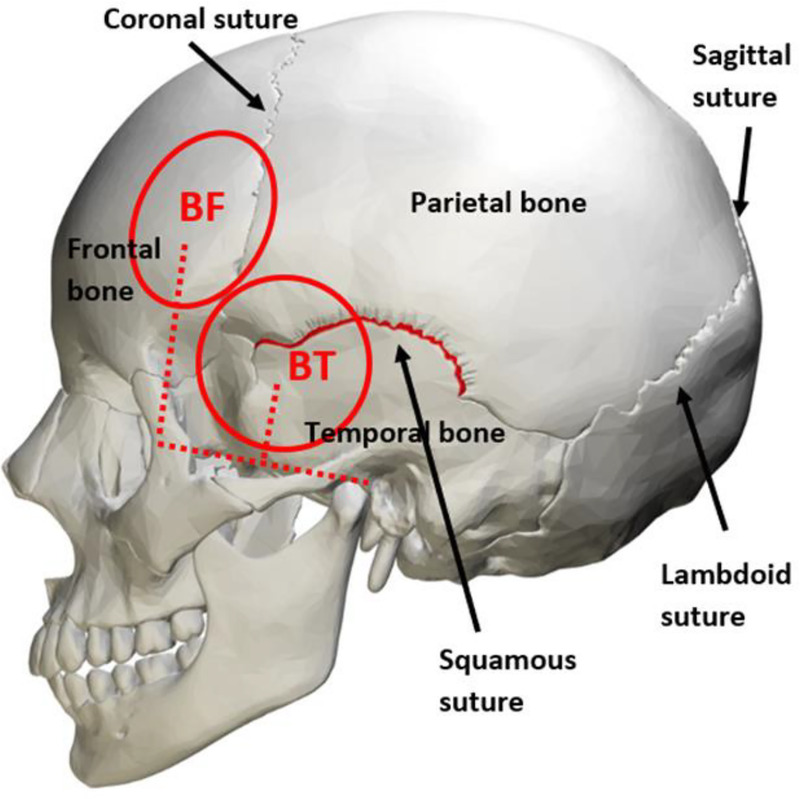
Major cranial sutures underlying ECT electrode placement. Human skull with the cranial sutures that lie below 2 ECT electrode placements; the BF—5 cm superior to the lateral canthus, and BT—3 cm superior to the midpoint of the lateral canthus and tragus. The red circles depict circular electrodes that are 5 cm in diameter. Image is licensed under Creative Commons Attribution 3.0 generic (Author OpenStax College from Wikimedia).

**FIGURE 2:**
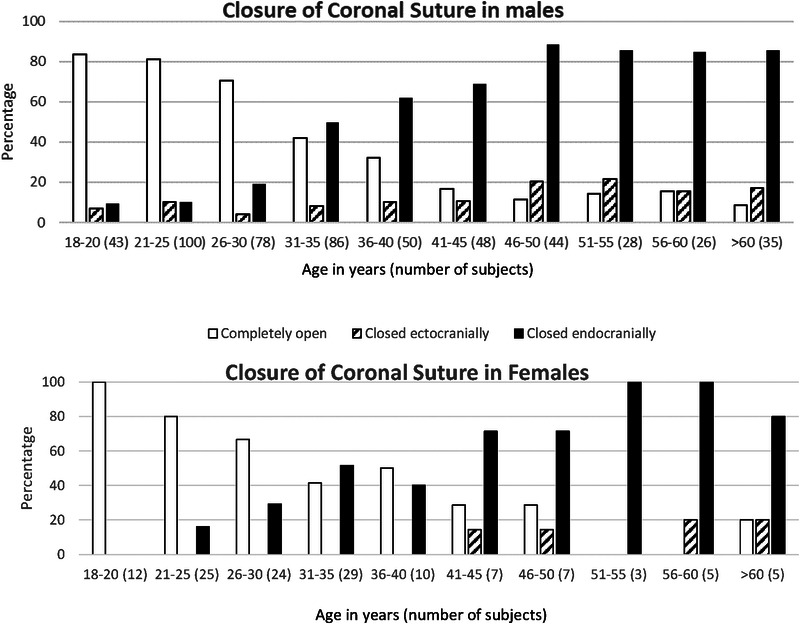
Closure of the right coronal suture with age from an autopsy study inmales and females.^16^ Ascardi and Nemeskeri technique (4 item scoring from 0 to 3) however partially closed sutures (1 and 2) were not included given low numbers.

There is a dearth of literature documenting the impact of cranial sutures on tES generated electric fields in the brain. Two decades ago, a review of ECT electrode placement postulated that electrode positioning near cranial sutures might influence clinical efficacy and cognitive side effects, given electrical resistance at cranial sutures is much lower than unperforated bone.^5^ This is relevant to routinely used ECT electrode placements given 2 sutures criss-cross the bitemporal (BT) electrode site and one suture lies under the bifrontal (BF) site (see Fig. 1). The frontal electrode placements in tDCS typically used to treat depression also overlie the coronal suture. In both cases, the electrical fields generated would travel through these sutures. Recent tDCS modeling of the F3 and F4 montage identified large interindividual variability in E-fields in targeted brain regions and between clinical and nonclinical populations^6^; however, it was limited given it modeled the skull as a single layer without sutures. Computational models can simulate electrical current flow with EEG montages and anatomically realistic head models are essential for their accuracy.

This review examines the importance of cranial sutures in understanding the electrical conductivity of the skull and for the computational modeling of tES. It will first review literature documenting the timing of cranial suture closure in adults, focusing on the coronal and squamous sutures relevant to tES. It will then consider the electrical conductivity of the skull components, comparing the cortical bone, spongiform bone and cranial sutures. Finally, a more accurate determination of cranial suture conductivity will be obtained through computational modeling and an imaging methodology will be assessed for quickly and accurately localizing the cranial sutures.

## METHODS

### Scoping Review of the Literature

A literature search was conducted using PubMed “Cranial Suture Closure AND forensic” to review articles that used cranial sutures forensically to age patients. Thirty-four studies were found. Nineteen studies were excluded, resulting in 15 studies. Exclusion criteria included the following: cranial suture other than coronal or squamous; studies with less than 50 subjects: case reports and letter to editors. Given squamous sutures are rarely used in forensic assessment and the more recent development of CT assessment of suture closure, a second specific search for “squamous suture closure AND CT” was conducted in PubMed (4 studies were found and 3 were excluded as they did not consider the squamous suture, resulting in 1 study). The search was repeated in SCOPUS (6 studies were found and 4 were excluded for not including the squamous suture, resulting in 2 studies). Finally, a literature search was conducted using PubMed “Skull [Title/Abstract] AND electrical conductivity [Title/Abstract].” This resulted in 23 studies, of which 16 studies were excluded resulting in 7 studies. Exclusion criteria included the following: studies not relevant to tES or EEG; studies that did not involve skull conductivity; children (under 18 yo) and animal studies.

### Computational Modeling to Determine Cranial Suture Conductivity

Computational simulations using COMSOL Multiphysics 6.1 finite element software of electrical current flow through bone plugs, 1.4 cm in size, using the same experimental method as outlined in the study by Tang et al.^7^ Two cylindrical saline layers of conductivity 1 S/m and 0.3 cm height (results did not vary when saline layer changed to 4 cm as per Tang et al^7^) were placed above and below the skull plugs, and a 1 V potential was applied between the top and bottom boundaries of the entire assembly. Total current flowing through the bone plugs was determined by integrating the normal component of current density across the top saline surface, which allowed overall resistance and conductivity of the plugs to be determined. Values of conductivity for compact bone, 0.0038 S/m was taken from Tang et al^7^ for standard compact skull plugs, and the value of conductivity for spongiform bone, 0.05 S/m, was taken from McCann et al^8^ with values shown in Table 1. Two skull plugs with sutures were simulated: 1) quasi compact (QC) skull plug with squamous suture and 2) QTC skull plug with coronal suture approximately reproducing the bone plug geometries of Tang et al^7^ including spongiform and compact bone regions (0.15 cm thick). The squamous suture was slightly narrower at 0.3 mm versus 0.4 mm for the coronal suture. Suture conductivity was varied in each case to achieve overall target plug conductivities demonstrated in Tang et al,^7^ finding a suture conductivity of 0.32 S/m able to match the target conductivity for both plugs. See Supplementary Material A for figures that detail the methodology, http://links.lww.com/JECT/A240.

**TABLE 1:** Conductivity of Skull Components

	Used in Our Model	McCann et al^8^—Weighted Mean (SD)
**Compact bone**	0.0038S/m^7^	0.0046 (0.0016)
**Spongiform bone**	0.05 S/m^8^	0.0497 S/m (0.0735)
**Suture**	**0.32 S/m—calculated**	0.0266 S/m (0.0239)

**FIGURE 3:**
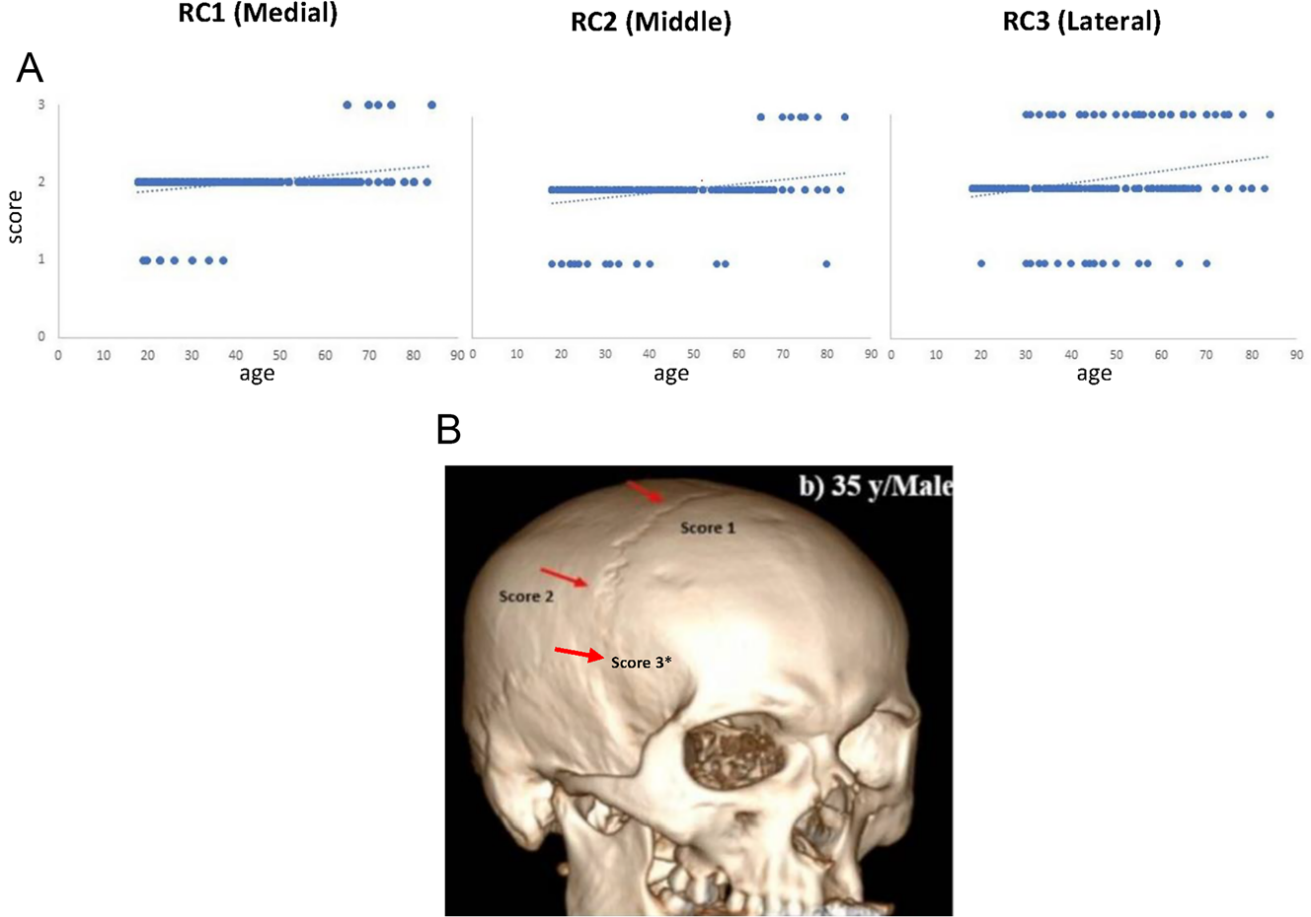
Closure of the right coronal suture with age assessed by head CT.^24^ A, Scatter plots showing linear regression models of age estimation using the right coronal suture segments (RC1—medial part, RC2—middle part, and RC3—lateral part (for score see “B”). B, Three-dimensional CT images of the skull representing the ectocranial scoring system: 1) incipient closure indicated by the evidence of bony bridging up to 50% closure; 2) significant closure indicated by the evidence of bony bridging greater than 50%; 3)* (not represented in image from referenced paper) obliteration with no trace remaining of the suture margins. Copyright 2023 (Creative Commons Attribution License).

### Volume Rendering of T1 and Fracture MRI DICOM Images Using 3D Slicer

DICOM data from a T1 MRI and Fracture MRI of a single subject were uploaded into 3D Slicer. 3D volume rendered images were produced and the preset “CT Lung” selected (or “CT Fat”). Sections of the 3D volume rendered images to demonstrate the cranial sutures were then isolated using the region of interest tool. See Supplementary Material B for figures that detail the methodology, http://links.lww.com/JECT/A241.

## RESULTS

### Cranial Suture Closure With Age

#### Coronal Suture

Cranial suture obliteration with age has been studied through forensic (autopsy) studies since the pioneering studies of Todd and Lyon in 1924 and 1925.^9,10^ However, a recent review concluded that this method was of questionable use in predicting age.^11^ Forensic studies of cranial suture closure with age commonly use 2 techniques: Meindl and Lovejoy (1985 which examines a single midpoint of the ectocranial aspect of the suture) and Ascardi and Nemeskeri (1970 which assesses the whole suture line and both ectocranial and endocranial aspects). A study in 175 dry skulls in Thailand (94 males and 81 females between 22 and 90 years) confirmed that the Ascardi and Nemeskeri technique is superior to Meindl and Lovejoy (a ~14-year variance vs 21-year variance),^12^ which has been previously demonstrated in other studies.^13,14^ This is likely because the endocranial suture closure correlates better with age.^15–17^ The most comprehensive of these studies was from India and included 665 adults (538 male and 127 female) of which 94% were aged between 18 and 60 years and 6% were over 60 years old.^16^ It demonstrated that coronal suture closure commences earlier in males than females and endocranial closure occurs before ectocranial closure. Ectocranial coronal suture closure has no correlation with age (see Fig. 2).

Other studies assessing the ectocranial closure of the coronal suture using the Meindl and Lovejoy technique demonstrated no correlation with age^12,13,18,19^ even when 1152 individuals were assessed using complex statistical methods.^20^

One of the reasons for the disparity of cranial suture closure with age is genetic factors. A study of 106 individuals during autopsy in Hungary (58 males and 43 females ranging in age from 18 to 97 yo) examined DNA and cranial suture closure data (using Meindl and Lovejoy's technique) and discovered that a single nucleotide polymorphism in MSH homeobox 1 (MSX1) correlated with ectocranial suture synostosis in adults.^21^ Much of the research into relevant genes relates to craniosynostosis, the premature closure of the cranial sutures in neonates, which occurs in approximately 1 in 2500 births. There have been over 60 mutations identified, the majority in fibroblast growth factor receptor genes. These influence the molecular signals between the approximating osteogenic fronts as they approach, either end-to-end (eg, coronal suture) or overlapping (eg, squamous suture). Other factors impacting cranial suture closure include Vitamin D deficiency (Rickets) and mechanical factors such as brain expansion and tensile stresses from inserted masticatory and neck muscles.^21^

Since 2010, CT has been used to assess suture closure in an attempt to more accurately represent age-related changes.^22^ A study in 2020 of 230 Chinese Han males aged from 23yo to 77 yo investigated 160 images from 16 suture segments in each individual and revealed that the variance of estimated age differed between the age groups (30–60 yo: less than 8 years; 20–30 yo and 60–70 yo: around 12 years; and for 70–80 yo: around 20 years).^23^ Another study in 2023 in India of 263 living individuals (183 males and 80 females aged from 18 to 84 years) used a 3-stage scoring system of the entire ectocranial aspect of the sutures (demonstrated in Fig. 3B)—this resulted in a variance of 14.74 years.^24^ The study demonstrated that the lateral aspect of the coronal suture closes earlier that the medial aspect (see Figs. 3A, B).

**FIGURE 4:**
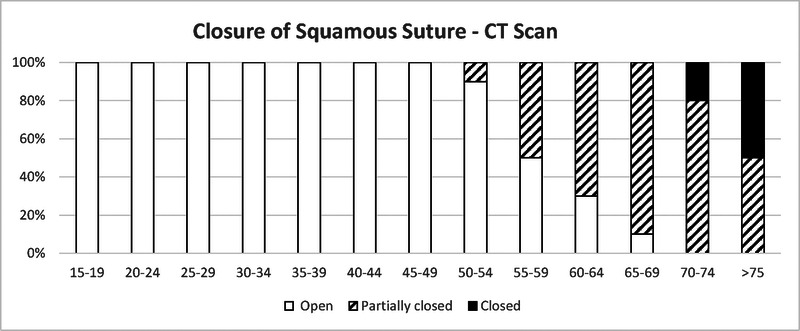
Closure of right squamous suture with age as assessed by Head CT in living subjects.^27^ The rating scale: completely open—clear joint space in whole length, partially closed—thinner and interrupted by partially closed areas, and closed—suture location is not recognizable.

These studies demonstrate that CT scans can assess suture closure in both living subjects and forensically and are more accurate in predicting the age of younger subjects. The coronal sutures tend to close endocranially with age and laterally prior to medially however the ectocranial aspect rarely completely closes.

#### Squamous Suture

The squamous suture is not used to forensically age skulls. The early studies of Todd and Lyon discovered that active closure of the squamous suture does not begin until 63 years of age. The posterior aspect does not close until 80 years while the anterior aspect never closes.^9,10^ A study of 211 dry skulls of adult male soldiers fallen in the Balkan Wars and WW1 (ranging in age from 1 to 60 yo revealed that only 1 case demonstrated bilateral obliteration of the squamous suture and 2 other cases unilateral obliteration).^25^ Two studies from India studied squamous suture closure using CT in living subjects. One included 100 individuals (60 males and 40 females with ages from 45 to 70 yo) and demonstrated that the squamous suture closes later than the lambdoid and parietomastoid sutures.^26^ The second study of 130 participants, with equal distribution of males and females ranging from 15 to 92 years of age also demonstrated the late closure of the squamous suture: it begins in the 5th decade and full closure does not start until the 7th decade. There was no demonstrated difference in the age of squamous suture closure between sexes (Fig. 4).^27^ These studies demonstrate that the squamous suture rarely closes with age.

**FIGURE 5:**
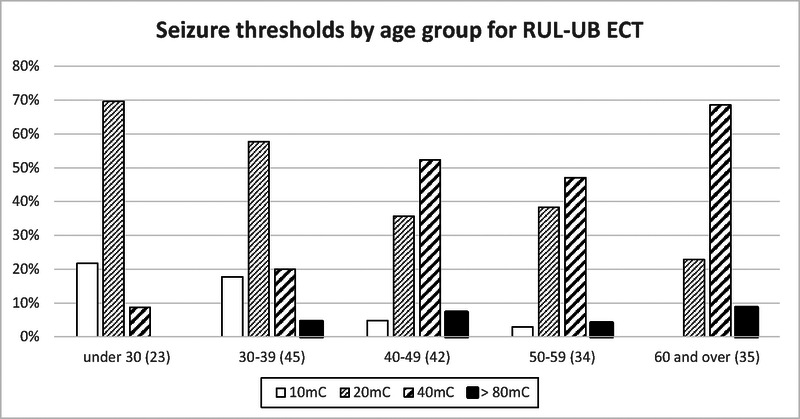
Distribution of seizure threshold for RUL-UB ECT with age. The number of male and female subjects is in parentheses.^28^

### Impact of Age on ECT Seizure Threshold

Loo and colleagues have previously demonstrated that older age is a predictor of higher seizure threshold for right unilateral ultrabrief (RUL-UB) pulse ECT (see Fig. 5).^28^ Atrophy of the brain, with associated increase in cerebrospinal fluid (CSF) which would shunt the electrical field, and white matter hyperintensities were postulated as reasons. There could also be a relationship between the increasing seizure threshold and age-related closure of the coronal suture and squamous suture, which lies near the electrode positions in RUL-UB ECT (right vertex and right temporal positions respectively). Given the high variability across individuals in age of suture closure, such as relationship could be verified in a study, which also assesses the extent of suture closure using T1 MRI DICOM images and 3D Slicer (see Supplementary Material B for details, http://links.lww.com/JECT/A241).

### Skull and Suture Conductivity

A study of a 2.2-cm circular skull plug taken from P3 (international 10–20 EEG site with no suture present) and soaked in normal saline demonstrated that a 3-layered representation, that is, 2 outer cortical bone layers and an inner spongiform layer (each with an isotropic value), is more accurate than a single layered anisotropic skull layer, when impedance is measured both radially (transverse) or tangential (parallel) to the skull.^29^ Despite this the skull is often represented as a single layer in computational models and to overcome this, a study assigned separate radial and tangential anisotropic values.^30^ Conductivity values of the skull have been determined in vitro (as above) and in studies in vivo, for example, using electrical impedance tomography (EIT) where 62 injection pairs were used to calculate the conductivity of the whole skull (5.5 mS) and compact bone (4.3 mS).^31^ Any increase in skull conductivity, for example via cranial sutures, will result in an increase in current density in the cortex during tES as the skull is the tissue with the lowest conductivity.^32^ However, we are not aware of any computational models of tES that include the cranial sutures.

A recent meta-analysis examined human head tissue conductivity from 56 studies, which used a variety of methodologies (in vitro, in vivo, and at various temperatures), resulting in a large range of measured values, as demonstrated in Table 1.^8^ The spongiform layer has increased conductivity due to fluid filled pores and cavities compared to the compact bone. An included study from 1993 took large 2.22-cm circular bone plugs from a single dried adult human skull at international 10–20 EEG sites and soaked them in 0.9% saline.^33^ A specimen taken from the F3 site (from left anterior frontal bone) was 6.2 mm thick, with substantial spongiform layer and the conductivity was 0.018 S/m. This contrasts with the T3M site (left temporal bone), which, despite being thinner (4.7 mm), had a reduced conductivity of 0.004 S/m.

**FIGURE 6:**
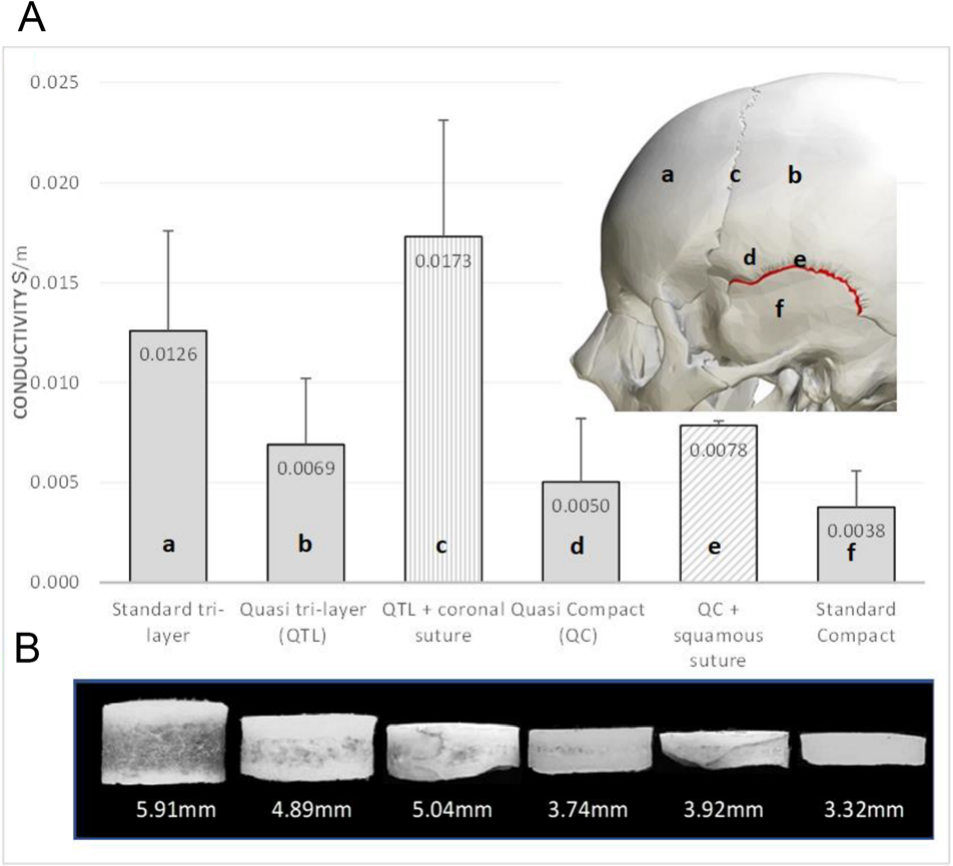
Conductivity of bone plugs taken from the skull from Tang et al.^7^ A, Graph representing the average conductivity (converted from resistance) of 6 bone plugs taken from approximate locations illustrated on the skull. B, Cross-sectional views of the 1.4 cm bone plugs with average thickness (Copyright 2008, IEEE). The vertical shaded column includes the coronal suture whereas the cross-hatched shaded column includes the squamous suture.

Tang et al^7^ took 388 1.4-cm bone plugs from skull flaps from 48 patients (20–74 yo with a mean age of 47.6 years) undergoing surgery for epilepsy, thus more representative of conductivity in vivo.^7^ Conductance was shown to increase proportionately to the percentage of spongiform bone and skull thickness. The coronal suture increased the conductance of the quasi tri-layer (QTL) bone by a factor of 2.5, whereas the squamous suture increased the conductance of the QC bone by a factor of 1.56 (see Fig. 6A).^7^ Figure 6B clearly depicts the coronal and squamous sutures in the bone plugs.

### EEG Source Imaging

**FIGURE 7:**
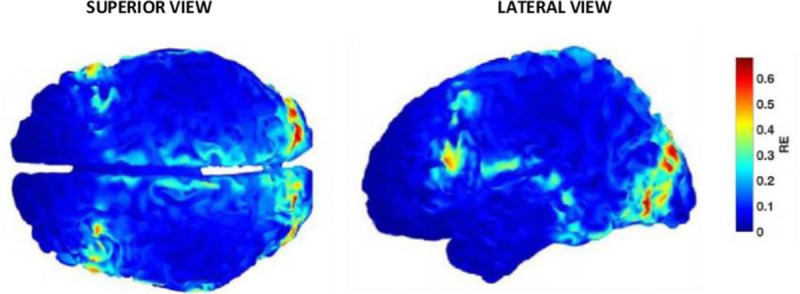
Error distribution for the EEG forward solution when omitting the cranial sutures.^36^ Relative error (RE) is presented, through a color scale, (RE is a ratio of the error compared to the measurement being taken) (Copyright IOP Publishing Limited 2019).

Source identification is an important objective for EEG and requires forward problem computations of the electric potential distributions on the scalp due to known sources. It is dependent upon realistic, anatomically correct head models with tissue conductivities accurately portrayed. Studies have demonstrated that ignoring emissary veins piercing the skull leads to local localization errors of 5–15 mm^34^ and that inclusion of CSF and white matter anisotropy is necessary for an accurate representation of the electric field inside the skull.^35^ In 2022, McCann and Beltrachini (authors of the meta-analysis described above^8^) created a high-resolution realistic head model and investigated the impact of varying cross-sectional proportions of spongiform bone, also including cranial sutures.^36^ The sagittal, coronal, and lambdoid sutures were assigned the average conductivity for dentate sutures of 0.0173 S/m and the squamous suture assigned a conductivity of 0.0079 S/m, values taken from the Tang et al^7^ study. It revealed that significant localized forward problem errors occurred along suture lines, with relative error up to 85% (see Fig. 7). It also demonstrated EEG—inverse problem inaccuracies of up to 4.14 cm across suture lines, which would lead to vastly different predicted sources in the brain for the measured EEG signal and potentially the removal of healthy brain tissue in epilepsy surgery.

### Computational Modeling to Estimate Cranial Suture Conductivity

**FIGURE 8:**
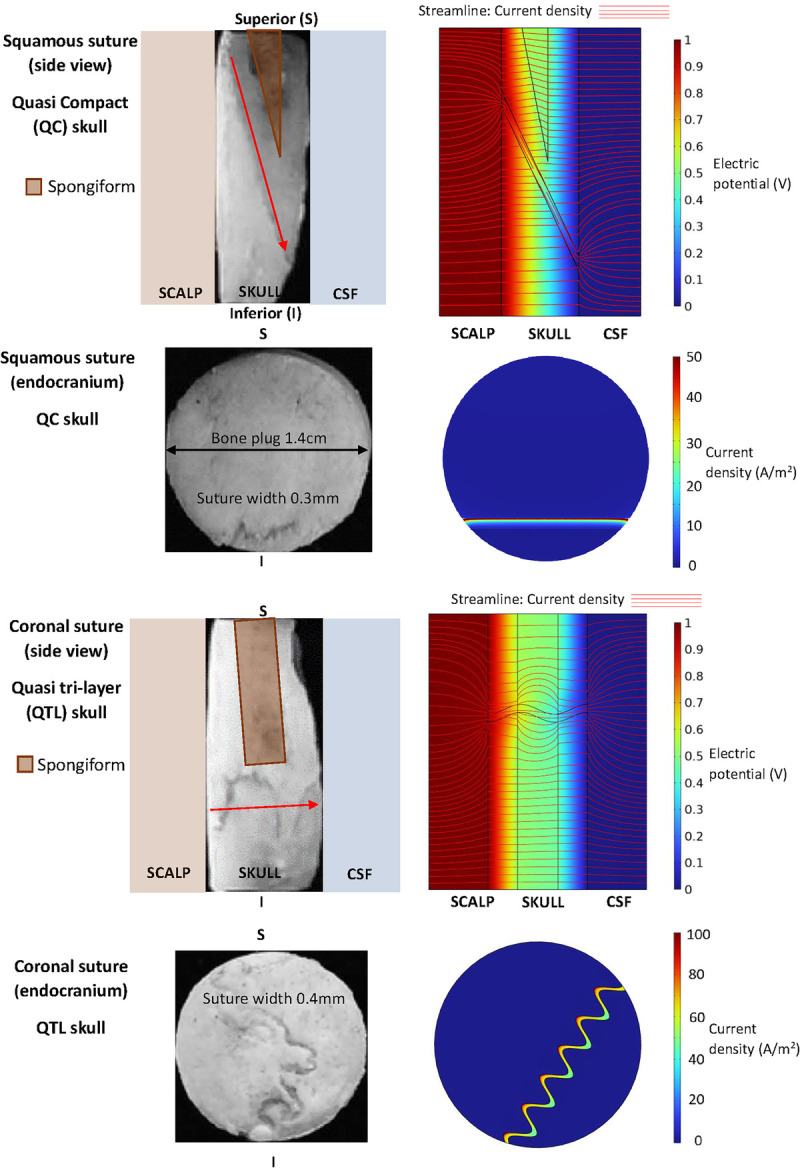
Computational simulation of electrical flow through bone plugs—using COMSOL Multiphysics finite element software and the experimental methods from Tang et al.^7^ Cylindrical saline layers (1 S/m) were placed each side of bone plugs and electrical current applied across 0.15 mm compact bone layers and variable spongiform bone geometries reproduced.

Bone impedance was measured across the sagittal suture in 100 Japanese individuals at autopsy (66 males and 34 females aged 6–89 yo) in an attempt to see if it correlated with age. The current was applied between 2 steel screws inserted 3 mm perpendicular into the skull, close to the margin of the sagittal suture, and demonstrated that the impedance increased up to 60 yo and then plateaued and started to decrease given postmenopausal bone changes.^37^ However, this does not represent the electrical current flow in tES, which runs perpendicular to the very resistant cortical bone of the skull. Given the much higher conductivity of the cranial sutures, they could theoretically form a conduit for the electrical current through to the CSF and brain. We conducted computational modeling of the electrical flow through 1.4-cm diameter bone plugs using COMSOL Multiphysics v6.1 (COMSOL AB, Sweden) finite element software and the experimental methods from Tang et al^7^ (see Fig. 8 and Supplementary Material A, http://links.lww.com/JECT/A240). Each component of the bone plug was given an isotropic conductivity (see Table 1). Given the whole plug conductivity was known, suture conductivity was determined as 0.32 S/m, which is much higher than the weighted mean in the cited meta-analysis (see Table 1). The squamous suture is narrower (0.3 mm) compared with the coronal suture (0.4 mm) and is linear and angled which reduces the current density on the endocranial surface of the skull. The modeling of the QC bone plug in Figure 8 demonstrates that the squamous suture has a much greater impact on conductivity than the spongiform bone represented, which had only a minimal impact on the streamline current density and did not alter the heat map on the endocranial surface of the skull (Fig. 8).

### Imaging of Cranial Sutures

**FIGURE 9:**
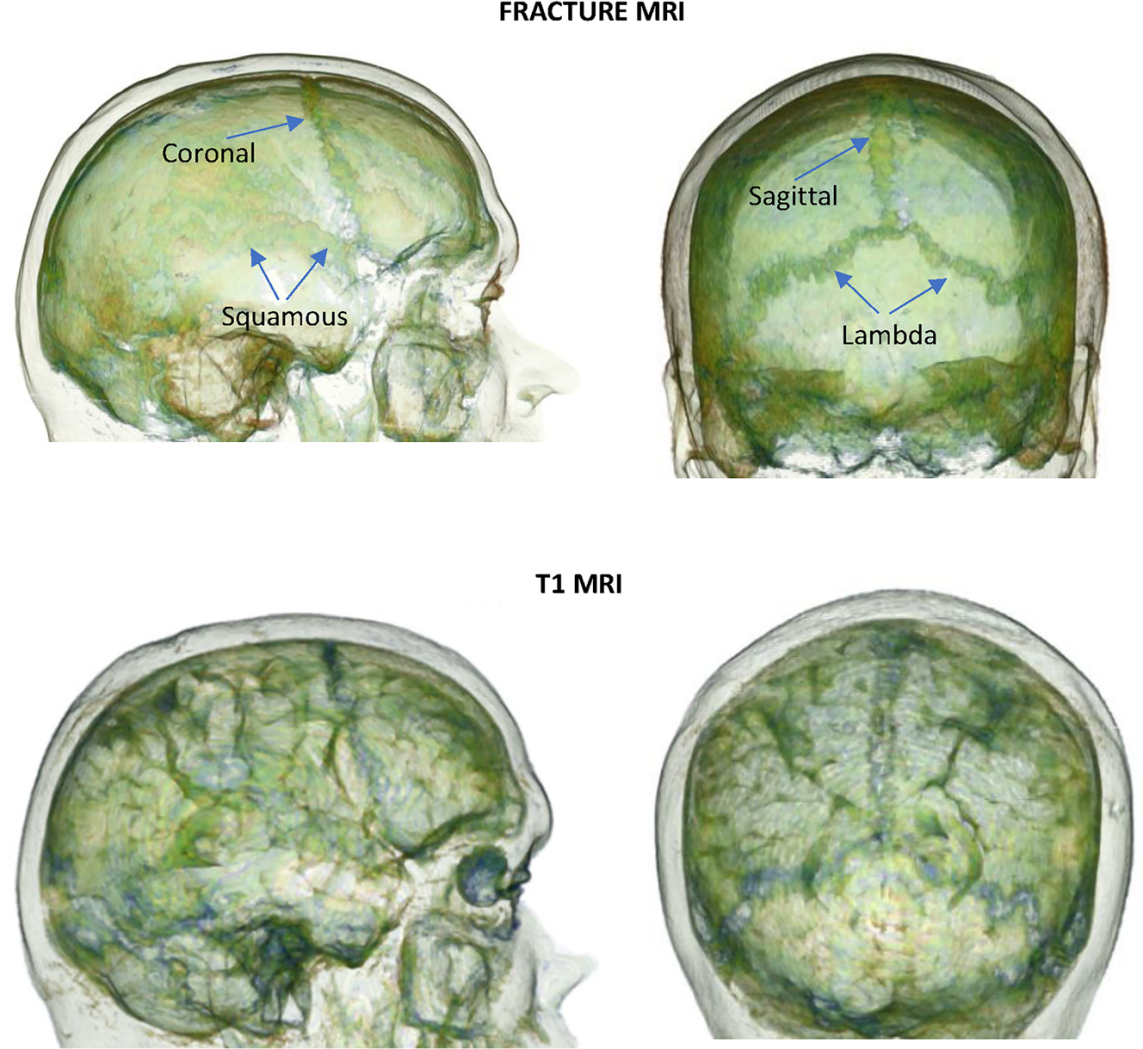
Volumetric rendering of T1 and FRACTURE MRI scan images—DICOM images of 45-year-old man (AC) uploaded to “3D Slicer v5.0.2” and volume rendering shift adjusted using the preset “CT-Lung” so the skull and sutures were visible. The sutures are clearly demonstrated in relation to soft tissue landmarks. **HREC number HC190222 (research MRI scanning for protocol development)**.

CT is currently the preferred modality for imaging the major cranial sutures as demonstrated in Figure 3B; however, consideration must be given to the radiation that patients are exposed to. Magnetic resonance imaging (MRI) scans do not expose patients to radiation but are more costly, take longer, and are less available. The short transverse relaxation times (T2) of bone and the low density of protons mean that imaging this tissue with MRI with adequate resolution to identify the inner and outer compact and spongiform layers is challenging. It requires newer MRI techniques such as ultra-short echo time^38^ or fast-field echo (FRACTURE) sequences to delineate bone. FRACTURE MRI provides more detail on bone structure than ultra-short echo time MRI and has similar accuracy to CT.^39^ Using freely available software, 3D Slicer (www.slicer.org), we demonstrated that the sutures can be easily identified from routine T1 weighted MRIs so more specialized MRIs are not necessarily required (see Supplemental Material B, http://links.lww.com/JECT/A241). The underlying brain cortex can also be seen in T1-weighted MRI images, which might assist with planning tES (see Fig. 9).

## DISCUSSION

Computational modeling has been increasingly used to study tES and EEG; however, precision is limited by the anatomical accuracy of realistic head models, which includes the cranial sutures. Our review firstly revealed that the timing of suture closure is variable between people and is not accurately reflected by age. We have demonstrated that cranial sutures can be quickly and precisely identified in relation to soft tissue landmarks by volumetric rendering of MRI scan images using 3D Slicer.

Sutures have higher electrical conductance compared with the skull. We have determined a cranial suture conductance of 0.32 S/m, which is much higher than for skull (0.004 S/m for compact skull and 0.013 S/m for standard trilayer^7^). It is therefore essential to incorporate sutures into computational modeling of tES given the coronal and squamous sutures are located under routinely used electrode placements for ECT and tDCS.

Including cranial sutures in tES modeling and EEG source imaging will improve the accuracy of current computational modeling techniques used in tES research. This may have important clinical applications. For example, with ECT, computational modeling is increasingly used to understand and optimize electrode placement.^40^ It could be used to optimize the much weaker tDCS E-field by targeting the electrodes over the more conductive cranial sutures and also facilitate the development of dose-response relationships for particular therapeutic outcomes. In EEG source imaging, it could lead to more precise results and avoid removal of healthy brain tissue in epilepsy surgery.

## References

[1] UK ECT Review Group. Efficacy and safety of electroconvulsive therapy in depressive disorders: a systematic review and meta-analysis. *Lancet*. 2003;361:799–808. 12642045 10.1016/S0140-6736(03)12705-5

[2] Sinclair DJ Zhao S Qi F, . Electroconvulsive therapy for treatment-resistant schizophrenia. *Cochrane Database Syst Rev*. 2019;3:CD011847. 30888709 10.1002/14651858.CD011847.pub2 PMC6424225

[3] Lefaucheur JP Antal A Ayache SS, . Evidence-based guidelines on the therapeutic use of transcranial direct current stimulation (tDCS). *Clin Neurophysiol*. 2017;128:56–92. 27866120 10.1016/j.clinph.2016.10.087

[4] Alam M, . A study on differences in the obliteration of cranial sutures and their clinical significance. *J Anat Soc India*. 2020;69:97–102.

[5] Swartz CM Nelson AI. Rational electroconvulsive therapy electrode placement. *Psychiatry (Edgmont)*. 2005;2:37–43. 21152159 PMC3000196

[6] Mizutani-Tiebel Y Takahashi S Karali T, . Differences in electric field strength between clinical and non-clinical populations induced by prefrontal tDCS: a cross-diagnostic, individual MRI-based modeling study. *Neuroimage Clin*. 2022;34:103011. 35487132 10.1016/j.nicl.2022.103011 PMC9125784

[7] Tang C You F Cheng G, . Correlation between structure and resistivity variations of the live human skull. *IEEE Trans Biomed Eng*. 2008;55:2286–2292. 18713698 10.1109/TBME.2008.923919

[8] McCann H Pisano G Beltrachini L. Variation in reported human head tissue electrical conductivity values. *Brain Topogr*. 2019;32:825–858. 31054104 10.1007/s10548-019-00710-2 PMC6708046

[9] Todd TW Lyon DW Jr. Endocranial suture closure. Its progress and age relationship. Part I. Adult males of white stock. *Am J Phys Anthropol*. 1924;7:325–384.

[10] Todd TW Lyon DW Jr. Cranial suture closure. Its progress and age relationship. Part II—ectocranial closure in adult males of white stock. *Am J Phys Anthropol*. 1925;8:23–45.

[11] Ruengdit S Troy Case D Mahakkanukrauh P. Cranial suture closure as an age indicator: a review. *Forensic Sci Int*. 2020;307:110111. 31901460 10.1016/j.forsciint.2019.110111

[12] Ruengdit S Prasitwattanaseree S Mekjaidee K, . Age estimation approaches using cranial suture closure: a validation study on a Thai population. *J Forensic Leg Med*. 2018;53:79–86. 29207328 10.1016/j.jflm.2017.11.009

[13] Wolff K Vas Z Sótonyi P, . Skeletal age estimation in Hungarian population of known age and sex. *Forensic Sci Int*. 2012;223(1–3):374.e1–374.e8. 10.1016/j.forsciint.2012.08.033 22975013

[14] Galera V Ubelaker DH Hayek LA. Comparison of macroscopic cranial methods of age estimation applied to skeletons from the Terry Collection. *J Forensic Sci*. 1998;43:933–939. 9729807

[15] Chawla H Shankar S Tyagi A, . Cranial vault suture obliteration in relation to age: an autopsy-based observational study. *Cureus*. 2023;15:e39759. 37398819 10.7759/cureus.39759 PMC10311457

[16] Sahni D Jit I Neelam, . Time of closure of cranial sutures in northwest Indian adults. *Forensic Sci Int*. 2005;148(2–3):199–205. 15739299 10.1016/j.forsciint.2004.06.002

[17] Schmitt HP Tamáska L. Forensic osteology. IV. Closure of the cranial sutures with special consideration of the question of age determination. *Z Rechtsmed*. 1970;67:230–248. 4320115 10.1007/BF02053747

[18] Boyd KL Villa C Lynnerup N. The use of CT scans in estimating age at death by examining the extent of ectocranial suture closure. *J Forensic Sci*. 2015;60:363–369. 25619969 10.1111/1556-4029.12683

[19] Xanthopoulou P Valakos E Youlatos D, . Assessing the accuracy of cranial and pelvic ageing methods on human skeletal remains from a modern Greek assemblage. *Forensic Sci Int*. 2018;286:266.e1–266.e8. 10.1016/j.forsciint.2018.03.005 29615347

[20] Konigsberg LW. Multivariate cumulative probit for age estimation using ordinal categorical data. *Ann Hum Biol*. 2015;42:368–378. 26190374 10.3109/03014460.2015.1045430

[21] Wolff K Hadadi E Vas Z. A novel multidisciplinary approach toward a better understanding of cranial suture closure: the first evidence of genetic effects in adulthood. *Am J Hum Biol*. 2013;25:835–843. 24123566 10.1002/ajhb.22459

[22] Harth S Obert M Ramsthaler F, . Ossification degrees of cranial sutures determined with flat-panel computed tomography: narrowing the age estimate with Extrema. *J Forensic Sci*. 2010;55:690–694. 20345795 10.1111/j.1556-4029.2010.01342.x

[23] Fan F Tu M Li R, . Age estimation by multidetector computed tomography of cranial sutures in Chinese male adults. *Am J Phys Anthropol*. 2020;171:550–558. 31891181 10.1002/ajpa.23998

[24] Akbar NJM Shekhawat RS Kanchan T, . Computed tomographic evaluation of cranial suture obliteration for age estimation in an Indian population. *Cureus*. 2023;15:e36160. 37065378 10.7759/cureus.36160 PMC10102175

[25] Nikolova S Toneva D Lazarov N. Squamous suture obliteration: frequency and investigation of the associated skull morphology. *Anat Sci Int*. 2021;96:42–54. 32591992 10.1007/s12565-020-00555-x

[26] Kohli K Aggarwal OP Mittal A. Age estimation of individuals beyond 45 years of age by CT scan of skull. *J Punjab Acad Forensic Med Toxicol*. 2019;19:125.

[27] Zanzrukiya KM Kumar L Bhalodiya AA. A cross sectional discriptive study of analysis of lambdoid and squamous sutures closure by Ct scan for age estimation. *Indian J Forensic Med Toxicol*. 2021;15:118–125.

[28] Gálvez V Hadzi-Pavlovic D Smith D, . Predictors of seizure threshold in right unilateral ultrabrief electroconvulsive therapy: role of concomitant medications and anaesthesia used. *Brain Stimul*. 2015;8:486–492. 25683317 10.1016/j.brs.2014.12.012

[29] Sadleir RJ Argibay A. Modeling skull electrical properties. *Ann Biomed Eng*. 2007;35:1699–1712. 17629793 10.1007/s10439-007-9343-5 PMC2496996

[30] Bai S Loo C Al Abed A, . A computational model of direct brain excitation induced by electroconvulsive therapy: comparison among three conventional electrode placements. *Brain Stimul*. 2012;5:408–421. 21962983 10.1016/j.brs.2011.07.004

[31] Fernandez-Corazza M Turovets S Luu P, . Skull modeling effects in conductivity estimates using parametric electrical impedance tomography. *IEEE Trans Biomed Eng*. 2018;65:1785–1797. 29989921 10.1109/TBME.2017.2777143

[32] Santos L Martinho M Salvador R, . Evaluation of the electric field in the brain during transcranial direct current stimulation: a sensitivity analysis. *Annu Int Conf IEEE Eng Med Biol Soc*. 2016;2016:1778–1781. 28268672 10.1109/EMBC.2016.7591062

[33] Law SK. Thickness and resistivity variations over the upper surface of the human skull. *Brain Topogr*. 1993 Winter;6:99–109. 8123431 10.1007/BF01191074

[34] Fiederer LDJ Vorwerk J Lucka F, . The role of blood vessels in high-resolution volume conductor head modeling of EEG. *Neuroimage*. 2016;128:193–208. 26747748 10.1016/j.neuroimage.2015.12.041 PMC5225375

[35] Bangera NB Schomer DL Dehghani N, . Experimental validation of the influence of white matter anisotropy on the intracranial EEG forward solution. *J Comput Neurosci*. 2010;29:371–387. 20063051 10.1007/s10827-009-0205-z PMC2912982

[36] McCann H Beltrachini L. Impact of skull sutures, spongiform bone distribution, and aging skull conductivities on the EEG forward and inverse problems. *J Neural Eng*. 2022;19. doi:10.1088/1741-2552/ac43f7 PMID: 34915464. 34915464

[37] Ishikawa N Suganami H Nishida A, . Utilization of bone impedance for age estimation in postmortem cases. *J Forensic Leg Med*. 2015;36:102–107. 26421720 10.1016/j.jflm.2015.09.006

[38] Krämer M Herzau B Reichenbach JR. Segmentation and visualization of the human cranial bone by T2* approximation using ultra-short echo time (UTE) magnetic resonance imaging. *Z Med Phys*. 2020;30:51–59. 31277935 10.1016/j.zemedi.2019.06.003

[39] Deininger-Czermak E Euler A Franckenberg S, . Evaluation of ultrashort echo-time (UTE) and fast-field-echo (FRACTURE) sequences for skull bone visualization and fracture detection - a postmortem study. *J Neuroradiol*. 2022;49:237–243. 34758365 10.1016/j.neurad.2021.11.001

[40] Steward B Bakir AA Martin D, . The left anterior right temporal (LART) placement for electroconvulsive therapy: a computational modelling study. *Psychiatry Res Neuroimaging*. 2020;304:111157. 32799057 10.1016/j.pscychresns.2020.111157

